# Next-generation sequencing approach for connecting secondary metabolites to biosynthetic gene clusters in fungi

**DOI:** 10.3389/fmicb.2014.00774

**Published:** 2015-01-14

**Authors:** Ralph A. Cacho, Yi Tang, Yit-Heng Chooi

**Affiliations:** ^1^Chemical and Biomolecular Engineering Department, University of CaliforniaLos Angeles, Los Angeles, CA, USA; ^2^Chemistry and Biochemistry Department, University of CaliforniaLos Angeles, Los Angeles, CA, USA; ^3^Plant Sciences Division, Research School of Biology, The Australian National UniversityCanberra, ACT, Australia

**Keywords:** filamentous fungi, secondary metabolites, gene clusters, next generation sequencing, genome mining

## Abstract

Genomics has revolutionized the research on fungal secondary metabolite (SM) biosynthesis. To elucidate the molecular and enzymatic mechanisms underlying the biosynthesis of a specific SM compound, the important first step is often to find the genes that responsible for its synthesis. The accessibility to fungal genome sequences allows the bypass of the cumbersome traditional library construction and screening approach. The advance in next-generation sequencing (NGS) technologies have further improved the speed and reduced the cost of microbial genome sequencing in the past few years, which has accelerated the research in this field. Here, we will present an example work flow for identifying the gene cluster encoding the biosynthesis of SMs of interest using an NGS approach. We will also review the different strategies that can be employed to pinpoint the targeted gene clusters rapidly by giving several examples stemming from our work.

## INTRODUCTION

Human health has been benefited from the secondary metabolites (SMs) produced by fungi. These small molecules, also known as natural products, include important clinical drugs like the antibiotic penicillins (Kardos and Demain, 2011), the cholesterol-lowering statins (Endo, 2010), the immunosuppressive cyclosporins (Britton and Palacios, 1982) and the antifungal echinocandins (Balkovec et al., 2014). Microbial SMs, including those from bacteria and fungi, continue to serve as important sources of molecules for drug discovery. For many decades, the fascinating and diverse structures of microbial SMs have inspired the organic chemists to embark on a quest to elucidate their biosynthetic pathways. Many basic insights into SM pathways were obtained by organic chemists using isotopic tracers during the 1950s (Bentley, 1999). The research shifted to the molecular biology of SM biosynthesis with the availability of tools for DNA cloning and sequencing. This is marked by several landmark papers, which described the molecular cloning of whole SM biosynthetic pathway on a contiguous stretch of DNA from actinomycete bacteria (Malpartida and Hopwood, 1984; Cortes et al., 1990; Donadio et al., 1991). Around the same period, the first fungal SM gene cluster, the penicillin biosynthetic gene cluster with the core non-ribosomal peptide synthetase (NRPS) gene encoding L-δ-(α-aminoadipoyl)-L-cysteinyl-D-valine (ACV) synthetase had been discovered in the fungus *Penicillium chrysogenum* (Díez et al., 1990). This is followed by the discovery of the terpenoid gene cluster encoding trichothecenes (Hohn et al., 1993), and polyketide gene clusters encoding aflatoxin/sterigmatocystin biosynthesis in *Aspergillus* sp. (Brown et al., 1996; Yu et al., 2004) and lovastatin biosynthesis in *A. terreus* (Kennedy et al., 1999). This hallmark trait of gene clustering was then observed in almost all other classes of fungal SM pathways including indole alkaloids and terpenoids (Keller et al., 2005). The tendency for the biosynthetic genes in microbial SM pathways to cluster on a chromosomal locus greatly accelerated the elucidation of enzymatic steps involved in biosynthesis of individual SM compounds using molecular biology approaches. Consequently, identification of the gene cluster that encodes the production of a given SM is now becoming the common first step toward elucidating the molecular and enzymatic basis for the biosynthesis of a given SM. Subsequent verification of the predicted gene cluster is often achieved via targeted deletion and/or heterologous expression of key biosynthetic genes. Further characterization of the biosynthetic pathway can be done by deletion of the individual biosynthetic genes in the cluster or reconstruction of the whole pathway in heterologous systems.

Targeted SM gene cluster discovery in fungi in the pre-genomic era is a tedious and time-consuming process. Traditionally, this was done by either complementation of blocked mutants by cosmid libraries (e.g. Mayorga and Timberlake, 1990; Hendrickson et al., 1999), or insertional mutagenesis followed by plasmid rescue from the blocked mutant (e.g., Yang et al., 1996; Chung et al., 2003). Both of these aforementioned methods rely on screening of blocked/complementation mutants, which work well for pigment compounds but can be cumbersome if the phenotype, cannot be easily observed or assayed. For example, in the pioneering work to identify lovastatin gene cluster, 6000 mutants were screened for restored lovastatin production by HPLC/TLC after transformation of *A. terreus* with a cosmid library (Hendrickson et al., 1999). Other methods for identifying key biosynthetic gene include antibody screening of cDNA expression library (e.g., Beck et al., 1990), differential display reverse transcriptase-PCR (e.g., Linnemannstons et al., 2002), suppression subtractive hybridization-PCR (O’Callaghan et al., 2003). However, these methods often lead to isolation of a single gene or partial gene cluster. Further cosmid library walking is required to obtain the whole gene cluster. Due to the relatively large genome size, genome scanning method, such as that demonstrated for discovery of enediyne antitumor antibiotic pathways in actinomycete bacteria (Zazopoulos et al., 2003), is less feasible for fungi.

Biosynthesis of polyketide SMs has been subjected to more intensive studies among other classes of SM pathways in fungi (Chooi and Tang, 2012). One of the earlier major advances in identification of fungal polyketide SM gene clusters is the development of degenerate primer PCR based on conserved ketosynthase (KS) domain of polyketide synthases (PKSs). The KS domain DNA fragments are then use as probes to identify the cosmid library clone carrying the whole or partial gene cluster. This method has been employed to localize the aflatoxin and fumonisin PKS genes (Feng and Leonard, 1995; Proctor et al., 1999). The subsequent primer sets developed to target KS domains of a non-reducing (NR-), partial-reducing (PR-), and highly-reducing polyketide synthases (HR-PKSs) are especially useful for localizing specific PKS gene clusters in cosmid libraries (Bingle et al., 1999; Nicholson et al., 2001). Pioneering work by Kroken et al. (2003) which performed a phylogenomic analysis of the PKS genes in the genomes of *Gibberella*, *Neurospora*, *Cochliobolus*, and *Botrytis* species revealed that fungal PKSs are likely to be derived from eight major lineages (Kroken et al., 2003). Nevertheless, despite the degeneracy of the primers and subdivision of fungal PKS genes in to different subclasses, some PKS genes in the genome can be still be missed by the degenerate primer PCR approach. Furthermore, it has to be bear in mind that, although most SM pathways are clustered on chromosome in fungi some of them can be split into two or three smaller subclusters, such as the pathways for dothistromin in *Dothistroma septosporum* (Chettri et al., 2013), tryptoquivaline in *A. clavatus* (Gao et al., 2011), echinocandin in *Emericella rugulosa* (Cacho et al., 2012), and prenylated xanthones in *A. nidulans* (Sanchez et al., 2011). There are also instances where the gene clusters of multiple SM pathways are intertwined together, such as the fumitremorgin, fumagillin, and pseurotin supercluster (Wiemann et al., 2013a). In such cases, the absence of whole genome sequence information can complicate the identification of the complete gene set for the target SM pathway.

Whole genome sequencing (WGS) of the target SM-producing fungus bypasses the need for the cumbersome library construction, screening, and chromosome walking. More importantly, the genome sequence can reveal the inventory of all the SM gene clusters in the producing fungus. Even though each fungus can harbor 30–50 SM gene cluster, the number is still finite and one of them must encode the SM of interest. For example, WGS of *G. zeae* with the Sanger sequencing method has allowed the systematic deletion of all 15 PKS genes in the fungal genome, which lead to identification of the gene cluster for zearalenone, aurofusarin, fusarin C and an unidentified black perithecial pigment (Gaffoor et al., 2005). This opens up the opportunities for detailed characterization of these pathways for zearalenone (Kim et al., 2005; Zhou et al., 2010; Lee et al., 2011), aurofusarin (Frandsen et al., 2006; Frandsen et al., 2011), and fusarin C (Niehaus et al., 2013). The development of next-generation sequencing (NGS) technologies in the last decade has dramatically lowered the cost for DNA sequencing and put the power of microbial WGS in the hand of individual laboratories (van Dijk et al., 2014). This technology revolution has energized the natural product research field and sparked some exciting NGS-based targeted SM gene cluster discovery projects in fungi (**Table 1**). Our work that used such NGS approach includes the discovery of SM clusters encoding viridicatumtoxin and griseofulvin (Chooi et al., 2010), tryptoquialanine (Gao et al., 2011), echinocandin (Cacho et al., 2012), asperlicin (Haynes et al., 2012), ardeemin (Haynes et al., 2013), and brefeldin (Zabala et al., 2014). Other studies that have taken the advantage of NGS have identified the SM clusters for fungal bicyclo[2.2.2]diazaoctane indole alkaloids (Li et al., 2012), equisetin (Kakule et al., 2013), and pneumocandin (Chen et al., 2013).

**Table 1:** Examples of biosynthetic gene clusters assigned to their respective compounds using next-generation sequencing technology.

Species	Sequencing Method	Characterized SM biosynthetic gene clusters	Reference
*Penicillium aethiopicum*	454	Griseofulvin	Chooi et al. (2010)
		Viridicatumtoxin	Chooi et al. (2010)
		Tryptoquialanine	Gao et al. (2011)
*Emericella rugulosa*	Illumina	Echinocandin B	Cacho et al. (2012)
*Aspergillus alliaceus*	Illumina	Asperlicin	Haynes et al. (2012)
*Aspergillus* sp. MF 297-2	Illumina	(-)-notoamide A	Li et al. (2012)
*A. versicolor* NRRL 35600	Illumina	(+)-notamide A	Li et al. (2012)
*P. fellutanum* ATCC 20841	Illumina	Paraherquamide A	Li et al. (2012)
*Malbranchea aurantiaca* RRC1813	Illumina	Malbrancheamide	Li et al. (2012)
*Glarea lozoyensis*	Illumina	Pneumocandin	Chen et al. (2013)
*A. fischeri*	Illumina	Ardeemin	Haynes et al. (2013)
*Fusarium fujikuroi*	454	Apicidin F	Niehaus et al. (2014)
*Eupenicillium brefeldianum*	454/Illumina	Brefeldin	Zabala et al. (2014)
*F. heterosporum*	Illumina	Equisetin and fusaridione A	Kakule et al. (2013)

Contemporary natural product research programs are now increasingly based on the understanding of the relationship between the SM molecules and the biosynthetic genes (Walsh and Fischbach, 2010). Although such studies are primarily motivated by the desire to understand the molecular and enzymatic basis of SM biosynthesis, the potential benefits and implications derived from such work are manifold. Firstly, the new chemical insights obtained in elucidating the metabolic pathway can be used to design more efficient total synthesis routes for complex natural products. Secondly, knowledge about the gene cluster can facilitate the metabolic engineering effort to increase the yield of useful SMs for commercial production (Pickens et al., 2011). The knowledge also forms the basis for generation of new SM analogs by mutasynthesis and combinatorial biosynthesis. A recent example is the combinatorial biosynthesis of benzenediol lactones (Xu et al., 2014b), which built on previous studies (Reeves et al., 2008; Wang et al., 2008; Zhou et al., 2010; Xu et al., 2013b). Novel biocatalysts useful for green chemistry and chemoenzymatic process development may also be discovered from SM pathways. For example, the characterization of the acyltransferase LovD in lovastatin pathway led to the development of green chemistry process for the semisynthetic cholesterol-lowering drug simvastatin (Xie et al., 2006, 2009; Gao et al., 2009; Xu et al., 2013a). Furthermore, the gene cluster information will be useful for knowledge-based genome mining for structurally-related compound in other fungi, e.g., the discovery of the immunosuppressive neosartoricin based on viridicatumtoxin biosynthesis genes (Chooi et al., 2012, 2013a). Importantly, these established links between genes and SMs are valuable knowledge that contributes to the overarching aims for (1) accurate prediction of SM structures based on DNA sequences and (2) rational design of biosynthetic pathways for synthesis of organic molecules. Finally, bridging the gaps between genes and molecules may facilitate our understanding of the natural functions of SMs using comparative genomics and transcriptomics tools (Chooi and Solomon, 2014).

It is important to note that a SM/natural product-motivated fungal genome sequencing project has different aims compared to the conventional WGS project coordinated by an international consortium of researchers associated with large sequencing centers. The major goal is to obtain the whole SM gene cluster that encode the targeted SM on a contiguous stretch of DNA sequence contig or scaffold. That means the completeness of the genome sequence coverage and whole-genome annotation is of lower priorities. The key question is how to rapidly narrow down and accurately pinpoint the correct SM cluster in the genome that encodes the production of a target SM. Getting an accurate initial prediction will significantly reduce the time spent on gene cluster verification. Below, we will provide the general work flow and guidelines for researchers who are considering adopting NGS technologies for targeted SM gene cluster discovery based on our own experience. We will also use several examples stemming from our work, two PKS pathways and two NRPS pathways, to illustrate the concepts and strategies.

## NEXT-GENERATION *DE NOVO* FUNGAL GENOME SEQUENCING AND ASSEMBLY

**FIGURE 1:**
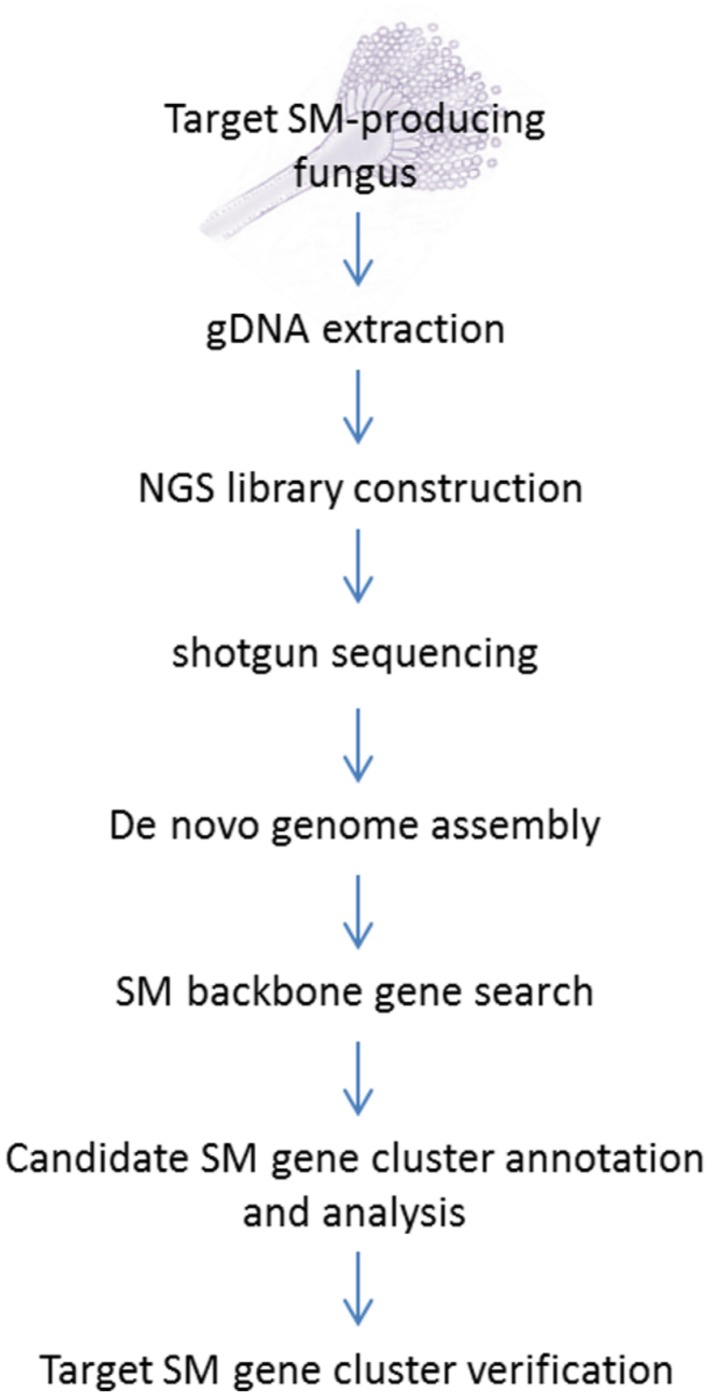
**Next-generation sequencing (NGS) workflow for targeted secondary metabolite (SM) gene cluster discovery**.

The standard NGS-based targeted SM gene cluster discovery work flow used in our laboratory is presented in **Figure 1**. The fungal strain acquired from culture collections or her sources are first verified for production of the targeted compound before its genomic DNA was sent for sequencing. A variety of culture media and conditions can also be tested to optimize the production of the target compound. Gene deletion or disruption followed by the detection of loss of target compound production is still the most common method for initial gene cluster verification. Alternatively, if genetic transformation is proven to be difficult on the fungus, expression of the backbone biosynthetic enzymes [e.g., PKS, NRPS, terpene synthase, or dimethylallyltryptophan synthase (DMATS)] in the candidate SM gene cluster in a heterologous system may be another way to verify the gene cluster.

The next thing to consider will be the choice of sequencing method. The choice will be mainly based on the cost, sequence quality, sequence read length, speed and project throughput (the number of strains to be sequenced together). There are currently several NGS technologies on the market suitable for *de novo* fungal WGS, with Illumina now dominating the market (Nowrousian, 2010; van Dijk et al., 2014). We have experience in both Roche/454 FLX Titanium and Illumina HiSeq2000, but other DNA sequencing platforms may work for this purpose as well. For an overview of the different latest NGS technologies, the readers are referred to van Dijk et al. (2014). The *de novo* sequencing of *P. aethiopicum* (synonym *P. lanosocoeruleum*) IBT5753 genome is one of the earliest examples of SM-motivated fungal WGS undertaken by individual laboratories, which used the Roche/454 FLX Titanium platform (Chooi et al., 2010).

The introduction of Illumina HiSeq2000 (now superseded by HiSeq2500) with ∼100 bp paired end (PE) reads dramatically reduced the cost of sequencing and increased the sequencing output. The shorter read length of HiSeq2000 is compensated by the deeper coverage and the paired-end nature of the Illumina reads. The Illumina PE reads allowed the assembly of longer scaffolds with gaps, which can be filled in later using routine PCR and Sanger sequencing if any of the scaffold harbor interesting SM gene cluster. Longer PE information can be obtained by generating mate pair libraries with larger insert size (e.g., 3 kbp or 5 kbp). Indeed, it has been recently demonstrated that good fungal genome assembly can be obtained on platform with shorter sequence reads (50 bp) like SOLiD using mate pair libraries (Umemura et al., 2013b).

We first used HiSeq2000 for sequencing of the brefeldin-producing *Eupenicillium brefeldianum* ATCC 58665 and the echinocandin-producing *E. rugulosa* (Cacho et al., 2012; Zabala et al., 2014). The amount of sequence data acquired for assembly of the ∼32 Mbp *E. rugulosa* genome is more than sufficient despite the shorter read length (N50 = 235 kbp). In fact, we can get equally good quality assembly using half the amount of data. Now, we routinely perform HiSeq2000 sequencing multiplexed for four fungal genomic samples per lane (∼200X depth of coverage each genome) and can routinely obtain good quality assembly for the purpose of SM gene cluster discovery. Using this arrangement, the cost of sequencing per fungal strain can be lower than the cost required for cosmid/fosmid library construction, screening, and chromosome walking, not to mention the significant time savings. With the use of mate pair libraries, more fungal genomic samples can likely be included into HiSeq2000 per lane yet still yield fine assembly. One recent study shows that the optimum sequencing depth for small bacteria to medium eukaryotic genomes with 2X 100 bp PE Illumina reads is 50-100X (Desai et al., 2013).

There is more than a dozen of software available for assembly of short reads generated by NGS, some are more memory intensive than the others, and there is differences in assembly speed as well. The popular ones including Velvet (Zerbino and Birney, 2008), SOAPdenovo (Li et al., 2010a), AllPATHS (Butler et al., 2008), and ABySS (Simpson et al., 2009). With optimization, some software may allow some small-medium size genomes to be assembled on a standalone workstation (Kleftogiannis et al., 2013). There are several studies that compare the efficiency and assembly quality of different assembly software (Lin et al., 2011; Zhang et al., 2011). We have experience mostly in using the SOAPdenovo assembler developed by BGI (Li et al., 2010a). Our SOAPdenovo assemblies were run on the UCLA Hoffman2 computer cluster. The assemblies can usually be completed with 32–128 GB memory requested from the cluster, depending on the genome size and the amount of input data. For the standard 2X 100 bp PE reads, we often use a *k*-mer size of 63 or 79 for the SOAPdenovo assembly and were able to obtain good results. The latest version, SOAPdenovo2, promised to improve memory efficiency and assembly quality, and more optimized for the longer Illumina reads (rather than the 35–50 bp reads from older Illumina platforms; Luo et al., 2012). Many NGS providers also provide service for sequence assembly with a fee.

## LOCATING THE TARGET SECONDARY METABOLITE GENE CLUSTER

**FIGURE 2:**
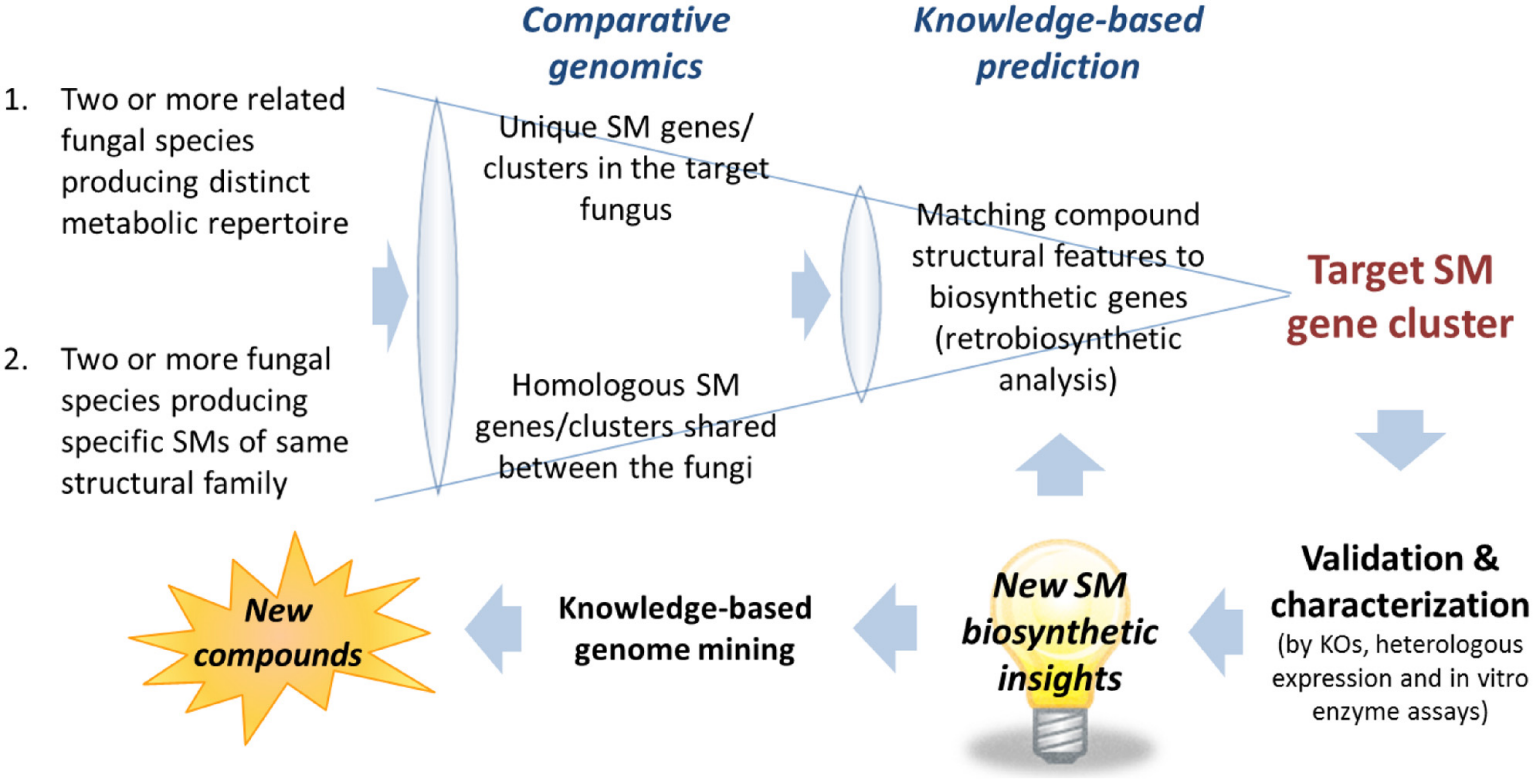
**Comparative genomics and knowledge-based prediction strategies for targeted SM gene cluster discovery.** New biosynthetic knowledge and insights into gene-to-molecular structure relationship would be useful for target SM gene cluster predictions and knowledge-based genome mining for discovery of novel SM compounds.

The first task after obtaining the scaffolds generated from the assembly is to narrow down the scaffolds containing the candidate SM gene clusters. Despite the enormous structural diversity, most fungal SMs can be divided into four major classes, polyketides, non-ribosomal peptides, terpenes, and indole alkaloids, based on the limited classes of carbon building blocks they derived from (Keller et al., 2005). Thus, an efficient way of narrowing down the scaffolds that may contain the target gene cluster is to search for genes encoding backbone biosynthetic enzymes that synthesized the specific class of compounds correspond to the target SM. This can be most easily achieved by performing a TBLASTN search against a database generated from the assembled fungal genome scaffolds using the “makeblastdb” command in the NCBI stand-alone BLAST application^1^. Depending on the class of SM, an arbitrary chosen conserved domain of the corresponding backbone enzymes can be used as a TBLASTN query. For example, a protein query sequence of a KS domain of PKS for polyketide SMs, an adenylation (A) domain of NRPS for non-ribosomal peptide SMs, terpene synthase for terpenoid SMs and DMATS for prenylated indole alkaloids. The TBLASTN will generate a list of scaffolds containing the SM gene clusters belongs the corresponding SM classes ranked based on homology to the query sequence. Alternatively, commercial bioinformatics software programs with intuitive graphic user interface (GUI) that support BLAST on local database are also available, e.g., CLC Genomics Workbench^2^ (CLC Bio) and Geneious^3^ (Biomatters). After locating the scaffolds containing gene clusters of the target SM classes, the number of candidate scaffolds can be further narrowed down with comparative genomics analysis (See Comparative Genomics Approach for Target SM Gene Cluster Prediction) followed by gene predictions and more in-depth knowledge-based bioinformatics analysis (see **Figure 2** and Section “Pinpointing Target SM Gene Cluster with Knowledge-Based Analysis – Retrobiosynthesis”).

For fungal gene predictions (the locations and exon–intron structures of genes on individual scaffolds) we routinely use FGENESH (Softberry; Solovyev et al., 2006), which can yield relatively accurate predictions for fungi. The web server allows direct submission of FASTA nucleotide sequences with gene-finding parameters trained using datasets from several fungal species^4^. Alternatively, AUGUSTUS also provides relatively accurate fungal gene prediction^5^ based on training sets from various fungal species (Stanke et al., 2004). Individual protein sequences predicted in a candidate scaffold are then submitted to NCBI BLASTP server for detailed conserved domain analysis (using the integrated NCBI Conserved Domain Search feature) and homologous sequence comparison. The EBI Interproscan also offers similar conserved domain prediction and protein functional analysis. As each gene cluster usually contain 3–15 genes (>20 genes in some cases), it is often not too time consuming when the number of candidate scaffolds has been narrowed down significantly. Several SM gene cluster prediction software programs have also been developed, which can aid this process considerably (see Pinpointing Target SM Gene Cluster with Knowledge-Based Analysis – Retrobiosynthesis).

### COMPARATIVE GENOMICS APPROACH FOR TARGET SM GENE CLUSTER PREDICTION

**FIGURE 3:**
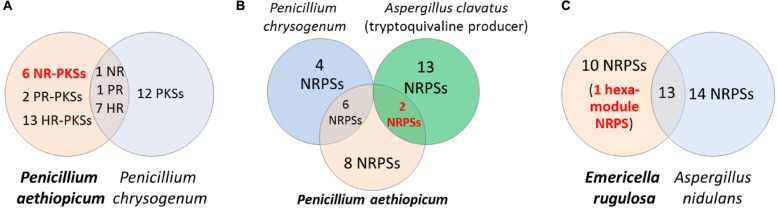
**Comparative genomics approach for discovery of SM biosynthetic gene cluster.** Comparison of the PKS genes in *Penicillium aethiopicum* and *P. chrysogenum* was utilized in searching for the griseofulvin and viridicatumtoxin gene cluster **(A)**. In order to narrow down possible candidates for the tryptoquialanine gene cluster in* P. aethiopicum*, NRPS genes that are non-orthologous to *P. chrysogenum* NRPS genes but are orthologous to the NRPS genes of the tryptoquivaline producer *A. clavatus* were found **(B)**. The echinocandin NRPS gene was found in *Emericella rugulosa* was found by searching for a hexamodule NRPS gene in *E. rugulosa* that is non-orthologous to *A. nidulans* NRPS genes (**C**).

Comparative genomics can be a useful approach for filtering of candidate gene clusters (**Figure 2**). The increasing number of sequenced fungal genomes in public databases, due in part to the lower sequencing cost enabled by NGS technologies, allows one to find a suitable genome of related sequenced organism for comparison with the organism of interest. Since the genomes of different organisms, even those belonging to the same genus, would encode different array of SMs, one can compare the SM backbone biosynthesis gene inventory of one organism with a closely related organism to rapidly narrow down the unique SM gene cluster. This approach is especially useful when there is pre-existing knowledge about the SM repertoire of the reference organism used for comparison. For example, in the search of SM gene clusters responsible for the biosynthesis of viridicatumtoxin and griseofulvin in *P. aethiopicum* (Chooi et al., 2010), we compared the PKS inventory of *P. aethiopicum* with that of *P. chrysogenum* based on prior knowledge from previous chemotaxonomy studies (Frisvad and Samson, 2004). Since it is known that *P. chrysogenum* produces neither viridicatumtoxin nor griseofulvin, we can first filter out the orthologous PKS genes shared between the two fungi (**Figure 3A**; Section Case Study A: Griseofulvin and Case Study B: Viridicatumtoxin). Similar the genome-wide comparison of NRPS genes in *A. nidulans* and *E. rugulosa* was used to narrow down the candidate echinocandin gene cluster (Cacho et al., 2012). It has to be bear in mind that the absence of report about the presence of a target SM compound in a specific species does not always correlate with the absence of the gene cluster in the genome as there can be strain-strain variation and many SM gene clusters could be transcriptionally silent. For example, TAN-1612, reported in another *A. niger* strain as BMS-192548 (Kodukula et al., 1995; Shu et al., 1995), is not detected in the sequenced *A. niger* ATCC 1015. However, the corresponding gene cluster can be identified and activated by transcriptional regulator overexpression (Li et al., 2011). The abilities to produce fumitremorgin by *A. fumigatus* (Kato et al., 2013), and gibberrellin and beauvericin by *G. fujikuroi* (Wiemann et al., 2013b), also vary from strain to strain. Nonetheless, such a comparative genomics approach had served as a useful first-pass filter in our hands, especially for fungal species that have been characterized chemotaxonomically, such as those species in the *Aspergillus* and *Penicillium* genera (Frisvad and Samson, 2004; Frisvad et al., 2007).

Conversely, comparative genomics can also be utilized for identifying the biosynthetic gene clusters of a target group of SMs bearing structural similarities but made by less-related fungal species (**Figure 2**). In this case, orthologous gene clusters in two organisms that are capable of synthesizing the same family of compounds are targeted. An example of the latter strategy is demonstrated in our work on tryptoquialanine/tryptoquivaline gene cluster identification in *P. aethiopicum*/*A. clavatus* (Gao et al., 2011; **Figure 3B**). The strategy is also well-demonstrated in the work by the Sherman group in investigating the biosynthesis of fungal bicyclo[2.2.2]diazaoctane indole alkaloids (-)- and (+)-notoamide A, paraherquamide A, and malbrancheamide A by *Aspergillus* sp. *MF297-2, A. versicolor* NRRL35600, *P. fellutanum* ATCC20841, and* Malbranchea aurantiaca* RRC1813, respectively (Li et al., 2012). More recently, NGS-driven comparative genomics of four *Stachybotrys* strains from two chemotypes producing either atranones or stratoxins have revealed two unique gene clusters, which possibly encode the biosynthesis of the two respective terpenoid-derived SMs (Semeiks et al., 2014). However, the identities of the two gene clusters are yet to be verified experimentally. Such comparative genomics approach is highly compatible with the high-throughput nature of Illumina sequencing as genomic samples from multiple fungal strains can be multiplexed on a single lane of an Illumina sequencer flow cell. In the case where the target SM gene cluster is split into more than one scaffold in one of the fungal genome assemblies, either due to disruption of assembly by repetitive sequence or the pathway is encoded by multiple loci, comparative genomics of the different strains can help localize the complete biosynthetic gene sets. This approach has facilitated the identification of genes for biosynthesis of tryptoquivaline in *A. clavatus*, which was separated into three genomic loci, by comparing with the tryptoquialanine-producing *P. aethiopicum* genome (Gao et al., 2011).

Lastly, one can use comparative genomics to make an educated guess on where the boundaries of the SM gene cluster are located. Oftentimes, SM gene clusters are flanked by syntenic blocks containing highly-conserved core genes (Machida et al., 2005; Tamano et al., 2008). Similarly, we have observed this trend in our studies toward the discovery of gene clusters encoding griseofulvin, viridicatumtoxin, cytochalasin, and tryptoquialanine. All four putative SM gene clusters are flanked by syntenic block of genes with high shared identity (>85%) in closely related ascomycetes species (Chooi et al., 2010; Gao et al., 2011; Qiao et al., 2011). In fact, a study reported a motif-independent bioinformatics approach for detection of SM gene cluster based on non-syntenic blocks in fungal genomes (Umemura et al., 2013a). One of the software that is useful for identifying and visualizing syntenic regions across multiple genomes is Mauve (Darling et al., 2004). While it does not guarantee that genes that are not highly conserved to a related organism are part of the cluster, this strategy is helpful toward minimizing the number of genes subjected to further functional analysis.

### PINPOINTING TARGET SM GENE CLUSTER WITH KNOWLEDGE-BASED ANALYSIS – RETROBIOSYNTHESIS

As mentioned above, gene predictions of individual scaffolds can be performed using software like FGENESH or AUGUSTUS after narrowing down the number of candidate SM gene clusters to a handful of scaffolds. Further detailed bioinformatics analysis of the individual SM gene clusters is then needed to identify the target gene cluster. Some software programs have been developed to aid SM gene cluster predictions. Two of the popular software programs available for prediction of SM gene clusters are antiSMASH (Medema et al., 2011; Blin et al., 2013) and SMURF (Khaldi et al., 2010). SMURF is specific for predicting SM gene clusters in fungal genomes, while antiSMASH can be used for both bacteria and fungi. Guidelines and detailed protocols for using these two and other related SM biosynthetic gene prediction programs can be found in Fedorova et al. (2012). SMURF requires a protein FASTA file and a gene coordinate file as input data. On the other hand, antiSMASH can accepts single nucleotide FASTA file and can be a very useful tool for getting an initial idea of the composition of SM gene cluster on each candidate scaffold along with gene annotation suggestions. Besides providing the predicted domain architecture of multi-domain backbone enzymes (i.e., PKSs and NRPSs), the smCOG (SM Cluster of Orthologous Groups) analysis module in antiSMASH also predicts the function of probable tailoring enzymes encoded in the cluster based on conserved domain analysis. Unfortunately, the substrate prediction function of antiSMASH is yet to be as useful for fungal SM clusters compared to bacterial ones. Moreover, intron prediction using antiSMASH is not as accurate compared to FGENESH, which can potentially hamper efforts toward heterologous expression of fungal SM biosynthetic enzymes in *E. coli* or yeast. With increasing number of established connections between fungal SM gene clusters and molecular structures, this feature is likely to improve in the future.

Nonetheless, a good understanding of the biochemistry of SM biosynthesis and knowledge about the relationship between biosynthetic genes and molecular structures is often needed to accurately pinpoint the SM gene cluster of interest. An excellent introduction to the common building blocks, enzymes, and biochemical reactions involve in SM biosynthesis is available (Dewick, 2009). The specific question that will be asked by the researcher is “what kind (and combination) of enzymes and precursors are likely to be involved in the biosynthesis of the target SM compound?” Such analytic-deductive approach is sometimes referred as “retro-biosynthetic analysis” where the SM structure are taken apart into simpler intermediates and precursors to help determine the enzymes and biological building blocks required for the target SM biosynthesis. Here, we will focus on the general strategies that can be adopted for pinpointing target PKS and NRPS gene clusters.

#### Polyketides

A great majority of fungal PKSs belong to the iterative type I PKSs whereupon the different PKS catalytic domains are juxtaposed on a single large polypeptide and the single set of PKS domains performs all the necessary catalytic activity during the biosynthesis of the polyketide. Fungal iterative type I PKSs are further classified into three major classes based on the degree of β-keto reduction performed by the PKS; namely the NR-PKSs, the PR-PKSs, and the HR-PKSs. The enzymology and classification of fungal iterative type I PKSs have been reviewed extensively (Cox, 2007; Crawford and Townsend, 2010; Chooi and Tang, 2012). As a general rule, aromatic polyketide compounds are synthesized by NR-PKSs, while HR-PKSs produce aliphatic compounds. PR-PKSs, on the other hand, has been shown to produce compounds lacking a phenolic hydroxyl group at the aromatic ring on the position where a β-keto group has been reduced to alcohol on the polyketide chain before cyclization, such as 6-methylsalicylic acid (Beck et al., 1990; Fujii et al., 1996) and (*R*)-mellein (Chooi et al., 2015). Thus, depending on the nature of the target polyketide compound, the number of candidate scaffolds can be further narrowed down by targeting NR-, PR-, or HR-PKS using corresponding KS domain. Querying the local BLAST database consist of the assembled genomic scaffolds with a corresponding KS domain will resulted in scaffolds containing the corresponding group of PKS genes appearing on top of the TBLASTN hit list. The specific group of PKSs encoded in the candidate scaffolds can be subjected to further scrutiny to pinpoint the PKS gene responsible for biosynthesis of the target SM. Type III PKSs, which present mainly in plants but can be found in some fungi as well, but are usually very limited in number (one or two) in most fungal genomes and are known to synthesize resorcylic acid-type compounds (Hashimoto et al., 2014).

In addition to the minimal PKS domains, a typical NR-PKS also contains the starter unit:ACP transacylase (SAT; Crawford et al., 2006); the product template (PT; Crawford et al., 2008, 2009). An NR-PKS could also contain a thioesterase/Claisen-like cyclase (TE/CLC; Fujii et al., 2001; Korman et al., 2010) or a terminal reductive domain (Bailey et al., 2007) for product release. Although examples of NR-PKSs utilize an in-*trans* releasing domain (Awakawa et al., 2009; Li et al., 2011) and NR-PKS that do not require any releasing domain or enzyme (Cacho et al., 2013) have been characterized as well. Of these aforementioned NR-PKS domains, the PT domain, which controls the first ring cyclization of the incipient reactive polyketide backbone to form the aromatic product, was shown to be useful in predicting the product of the NR-PKS. In the work by Li et al. (2010b), sequences of PT domains from characterized NR-PKS were subjected to phylogenetic analysis resulting in the grouping of the PT domains in accordance to their respective cyclization regiospecificity. The authors validated the model by demonstrating the previously uncharacterized PT domain of *An03g05440* from *A. niger*, predicted to catalyzed a C2-C7-type cyclization, does indeed perform the expected cyclization mode in a chimeric PKS *in vitro* system. Ahuja et al. (2012) has made similar observation for NR-PKSs in *A. nidulans.* Such PT domain analysis has facilitated the identification of viridicatumtoxin and TAN-1612 gene cluster (Chooi et al., 2010; Li et al., 2011).

Unlike NR-PKSs, HR-PKSs utilize the triad of the β-keto reductive domains ketoreductase (KR), dehydratase (DH), and enoylreductase (ER) domains to introduce complexity in the incipient polyketide during each extension cycle. However, in contrast to NR-PKSs, there is a lack of in-depth bioinformatic studies to predict the products of uncharacterized HR-PKS. While more studies in deciphering the biosynthetic rules programmed within these tailoring domains is needed to construct an accurate model of predicting HR-PKS product, important clues can be garnered about the possible product of the HR-PKS using rudimentary bioinformatic analysis. For instance, conserved domain prediction analysis tools can help reveal the presence of in-*cis* polyketide tailoring domains in the HR-PKS protein sequence such as a *C*-methyltransferase domain that would indicate α-methylation during one or more polyketide extension cycle. This feature has been exploited for identification of the PKS gene encoding the biosynthesis of the tetraketide side chain of squalestatin in a genomic FNA library using *C*-methyltransferase domain sequence probe (Cox et al., 2004). High sequence similarity and proximity of phylogenetic relationship with a characterized HR-PKS could, to a limited extend, suggest that the unknown PKS produce similar chain length and structure (Kroken et al., 2003).

Fungi use division of labor between NR-PKS and HR-PKS to generate compounds with an aromatic portion and highly-reduced aliphatic portion respectively (Chooi and Tang, 2012). The linear aliphatic chains generated by HR-PKSs are often used as starter units for NR-PKSs. Compounds generated by such NR/HR two-PKS systems include the resorcylic acid family of compounds (Zhou et al., 2010; Xu et al., 2013b) and asperfuranone (Chiang et al., 2009). The linear aliphatic acyl chain may also be attached to an aromatic polyketide portion as an ester, such as in the case of azanigerone (Zabala et al., 2012) and chaetoviridin (Winter et al., 2012). Thus, such structural features, if present in the target SM, will be a useful tell-tale for predicting the target SM gene cluster as there is limited (one or two) such two-PKS gene cluster(s) in filamentous fungal genomes surveyed to date. SM gene clusters encoding hybrid polyketide-nonribosomal peptide compounds (Boettger and Hertweck, 2013), such as cytochalasins (Qiao et al., 2011; Ishiuchi et al., 2013), equisetin (Kakule et al., 2013), pseurotin (Maiya et al., 2007), and fusarin C (Niehaus et al., 2013) can be identified easily in a genome as well as there is usually one or two PKS-NRPS hybrid gene(s) in most ascomycete fungal genomes. Further analysis of the tailoring enzymes encoding in the vicinity of these PKS genes can help pinpointing the exact target SM gene cluster (see Tailoring Enzymes).

#### Non-ribosomal peptides

Unlike iterative fungal PKSs, most fungal NRPSs are modular. Like the bacterial counterparts, fungal NRPSs are assembly-line-like protein complexes arranged in functional units known as modules (Finking and Marahiel, 2004, Strieker et al., 2010). Each NRPS module is minimally comprise of three domains: an adenylation (A) domain that selects and activates the amino acid (aa) substrate of the module, a thiolation (T) or peptidyl carrier protein (PCP) domain that serves as a covalent tether for the aa substrate or the growing peptide chain and a condensation (C) domain that catalyzes the peptide bond formation. One notable characteristic of NRPS enzymology is the ability of NRPSs to incorporate non-proteinogenic aa into the NRPS product (Walsh et al., 2013). In addition, NRPSs typically follow the collinearity rule such that the substrate specificity, the number and the linear arrangement of the module within the assembly determines the composition of the NRPS product. Thus, in most cases, the candidate NRPS-encoding genomic scaffolds can be narrowed down easily by first matching the number of modules in the NRPSs to the number of aa residues linked by peptide bonds in the target non-ribosomal peptide product. The presence of the in-line tailoring domain in the NRPS assembly line such as an epimerization (E) or N-methylation (M) domains can further indicate that the NRPS product undergoes respective modification.

There are some exceptions to the co-linearity rule for some fungal NRPSs, in which certain domains or modules on these NRPSs are used iteratively. Notable examples are the fungal siderophore NRPSs, which involved the iterative use of one of the A domains (activating an identical aa residue more than once in a complete NRPS catalytic cycle). These NRPSs harbor additional T–C partial modules that extend the non-ribosomal peptide products beyond the number of complete A–T–C modules in the NRPSs (Schwecke et al., 2006; Johnson, 2008). There are also other non-canonical NRPSs, such as those that synthesize fungal cyclooligomer depsipetides (Glinski et al., 2002; Xu et al., 2008; Sussmuth et al., 2011) and the recently identified fungisporin NRPS from *P. chrysogenum* (Ali et al., 2014).

Pioneering studies by the Marahiel group led to the development of the A domain 10 aa code, which aided in prediction of the substrate specificities of adenylation of uncharacterized bacterial NRPSs via sequence alignment (Conti et al., 1997; Stachelhaus et al., 1999). Other algorithms that predict adenylation domain substrate specificity and online servers that utilized these algorithms were subsequently developed and are now in widespread use for preliminary bioinformatic characterization of NRPS genes (Challis et al., 2000; Röttig et al., 2011). However, the utility of the A domain 10 aa code and NRPS analysis software programs for prediction of fungal NRPS domain is still limited for fungi compared to bacteria, as there remains a lack of sequence-substrate specificity relationship for fungal NRPSs. Nonetheless, the new NRPSpredictor2 software incorporates A domain substrate specificity prediction for fungal NRPSs and can be a useful starting point (Röttig et al., 2011). Some progress have been made in this area for the A domain 10 aa codes for anthranilate (Ames and Walsh, 2010), α-keto acids (Wackler et al., 2012), and L-tryptophan (Xu et al., 2014a). Phylogenetic and structural analysis of fungal A domains may also yield some insights into the possible substrate of the A domain and the structural class of the final product (Ames and Walsh, 2010; Bushley and Turgeon, 2010). For instance, characterization of the anthranilate-activating fungal A domain in fumiquinazoline F biosynthesis (Ames and Walsh, 2010) led to the discovery of the gene clusters of fungal anthranilic acid-containing non-ribosomal peptides (Gao et al., 2011; Haynes et al., 2012, 2013).

#### Tailoring enzymes

Since genes for the biosynthesis of SMs are typically clustered together, the types of tailoring reaction the polyketide or non-ribosomal backbone undergoes can be surmised based on the type of functional domains encoded in vicinity of the PKS or NRPS gene, respectively. Essentially, if the backbone biosynthetic gene analysis narrowed down the target SM gene cluster to a couple of possibilities, the combination of tailoring enzymes that matches the SM structure would allow one to confidently pinpoint the right cluster. Tailoring enzymes involved in functional-group transfer such as methyltransferases, acyltransferases, prenyltransferases, and halogenases are especially helpful in correlating a gene cluster to its corresponding compound since there is little ambiguity on what type of reaction these enzymes catalyzes. As described in Section 4 below, the co-localization of multiple methyltransferase genes with an NR-PKS gene was critical toward the discovery of the griseofulvin gene cluster, while the presence of a prenyltransferase gene and a methyltransferase gene flanking an NR-PKS gene pinpointed the correct viridicatumtoxin gene cluster (Chooi et al., 2010). Genes encoding redox enzymes, such as flavoenzymes, cytochrome P450s, and non-heme iron oxygenases, can suggest that the product of the cluster undergoes oxidative transformation. However, their role in biosynthesis can be ambiguous as these enzymes can catalyze diverse redox reactions including hydroxylation, epoxidation, oxidative cleavage, and rearrangement. This is illustrated by our work in functional elucidation of the redox enzymes in viridicatumtoxin, tryptoquialanine, and echinocandin B pathways. As described below, some of the functions of these redox enzymes turned out to be quite surprising. Thus, caution is advised on inferring the function of genes for redox enzymes based on conserved domain analysis, especially in cases where there is no known characterized enzyme that share high sequence similarity with the enzyme of interest.

## EXAMPLES ILLUSTRATING THE STRATEGIES FOR TARGETED SM GENE CLUSTER DISCOVERY

### CASE STUDY A: GRISEOFULVIN

Griseofulvin is an antifungal polyketide made by several different *Penicillium* species (Frisvad and Samson, 2004). To identify the griseofulvin gene cluster, a curation of the PKS genes in *P. aethiopicum* was performed. Using a local BLAST search of the genome using an arbitrary KS as a query, 30 putative intact PKS genes were found within the genome of *P. aethiopicum*. Comparison of the PKS genes in *P. aethiopicum* and the closely related species *P. chrysogenum* revealed that the former contains six NR-PKS genes that are not orthologous to *P. chrysogenum* NR-PKS genes (van den Berg et al., 2008; Chooi et al., 2010). Since *P. chrysogenum* was not known to produce griseofulvin, it was inferred that one of the six non-orthologous NR-PKS genes in *P. aethiopicum* was responsible for the biosynthesis of griseofulvin (**Figure 3A**).

**FIGURE 4:**
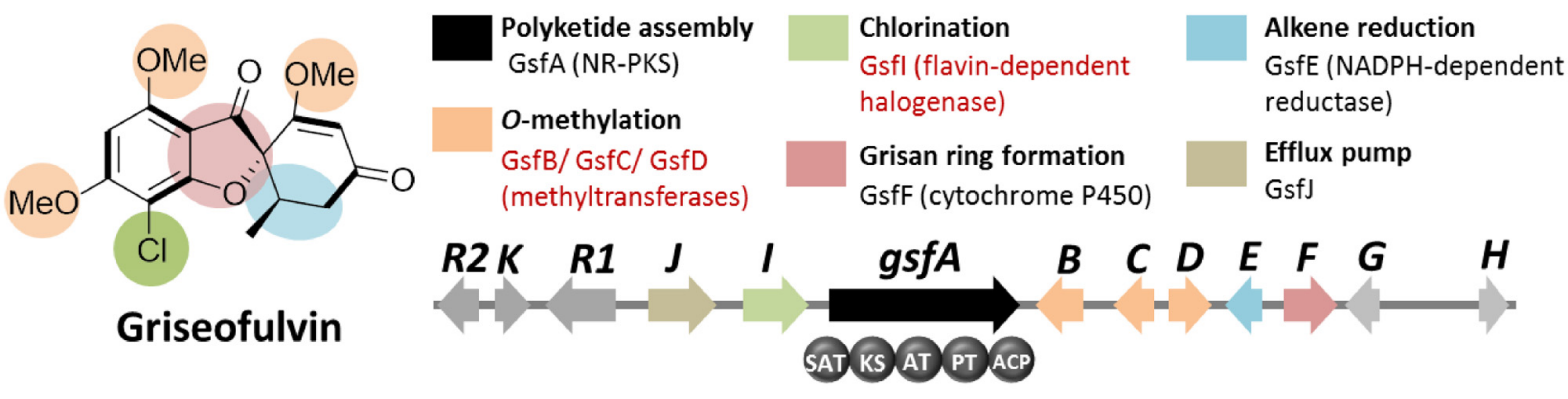
**Griseofulvin biosynthetic gene clusterin *P. aethiopicum*.** Shown are the roles of the genes in the biogenesis of the compound. The non-reducing polyketide synthase (NR-PKS) gene *gsfA* is found via comparison of PKS genes in *P. aethiopicum* and *P. chrysogenum*. Two structural features, *O*-methylation and chlorination (in red text), were used as criteria to find the candidate gene clusters.

Based on the distinct structural features of griseofulvin, the presence of multiple methyltransferase genes as well as a chlorinase gene in the gene cluster encoding griseofulvin is expected (**Figure 4**). Using these search criteria, the *gsf* gene cluster, the candidate gene cluster for the biosynthesis of the compound, was found. Along with the expected three *S*-adenosyl-methionine (SAM) methyltransferase genes (*gsfB-D*) and a flavin-dependent halogenase gene (*gsfI*) flanking the NR-PKS gene* gsfA*, genes for the two redox enzymes GsfE and GsfF were also found within the cluster (**Figure 4**). Deletion of the *gsfA* gene led to loss of production of griseofulvin in *P. aethiopicum* and thus confirming the role of the *gsf* cluster in the biosynthesis of the compound (Chooi et al., 2010). A subsequent paper by our group revealed the function of the genes in the cluster as well as the regioselectivity of the *O*-methyltransferases GsfB, C, and D (Cacho et al., 2013). A combination of gene deletion studies and reconstitution of the enzymatic reaction in the same study also revealed the role of GsfF and GsfE in the grisan ring formation and cyclohexadienone reduction, respectively.

### CASE STUDY B: VIRIDICATUMTOXIN

**FIGURE 5:**
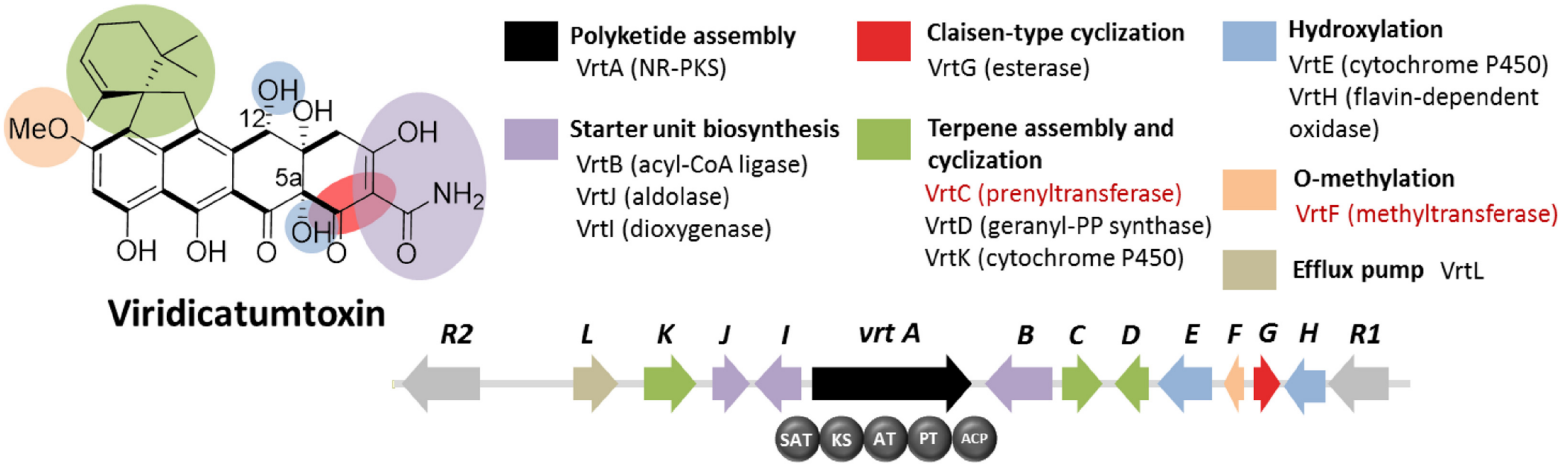
**Viridicatumtoxin biosynthetic gene cluster in *P. aethiopicum*.** As with the case for the griseofulvin, the NR-PKS gene *vrtA* is non-orthologous to PKS genes in *P. chrysogenum*. The presence of the prenyltransferase gene *vrtC* and the *O*-methyltransferase gene *vrtF* (in red text) led to the identification of the gene cluster.

Viridicatumtoxin is another aromatic polyketide synthesized by *P. aethiopicum*. It contained a naphthacenedione core reminiscent of the core of the bacterial tetracyclines. Moreover, viridicatumtoxin also contained a cyclized geranyl moiety (**Figure 5**), indicative of the possible presence of a prenyltransferase gene within the cluster. As in the case of griseofulvin, viridicatumtoxin was not known to be produced by *P. chrysogenum* and likewise, one of the non-orthologous NR-PKS genes in *P. aethiopicum* was presumably responsible for the biosynthesis of the compound (**Figure 3A**). In addition to the aforementioned cyclized terpene group, viridicatumtoxin also contained an *O-*methyl group similar to what was found in griseofulvin. Using these two structural features as “landmarks,” the viridicatumtoxin biosynthetic (*vrt*) gene cluster is localized on a scaffold (Chooi et al., 2010). Flanking the NR-PKS gene *vrtA* are genes encoding a farnesyl diphosphate synthase analog (geranyl diphosphate synthase), a prenyltransferase gene *vrtC* and a SAM-dependent *O*-methyltransferase gene *vrtF*; all three are in agreement with the two distinctive chemical features found in the compound. Deletion of the NR-PKS gene *vrtA* confirmed the role of the cluster in the biogenesis of the compound (Chooi et al., 2010).

Subsequent characterization of the prenyltransferase VrtC opened the doors toward the discovery of new fungal monoterpenoid biosynthetic gene cluster through genome mining (Chooi et al., 2012). In addition to the aforementioned genes, other genes encode five redox enzymes (*vrtE*, *vrtG*, *vrtH*, *vrtI*, and *vrtK*), a PLP-dependent threonine aldolase gene *vrtJ* and an acyl-CoA ligase gene *vrtB* were also found in the *vrt* gene cluster (Chooi et al., 2010). It was later found that VrtG and other related dimanganese-dependent thioesterase catalyze the Claisen-type cyclization in viridicatumtoxin and other fungal naphthacenediones (Li et al., 2011). Surprisingly, the cytochrome P450 VrtK, instead of a terpene cyclase, was shown to mediate the cyclization of the geranyl moiety found in viridicatumtoxin to afford a spirobicyclic structure fused to the tetracyclic core (Chooi et al., 2013b). Additionally, later studies revealed the role of VrtE and VrtH in the hydroxylation in the 5 and 12a positions in viridicatumtoxin, respectively (Chooi et al., 2013a,b).

### CASE STUDY C: TRYPTOQUIALANINE

**FIGURE 6:**
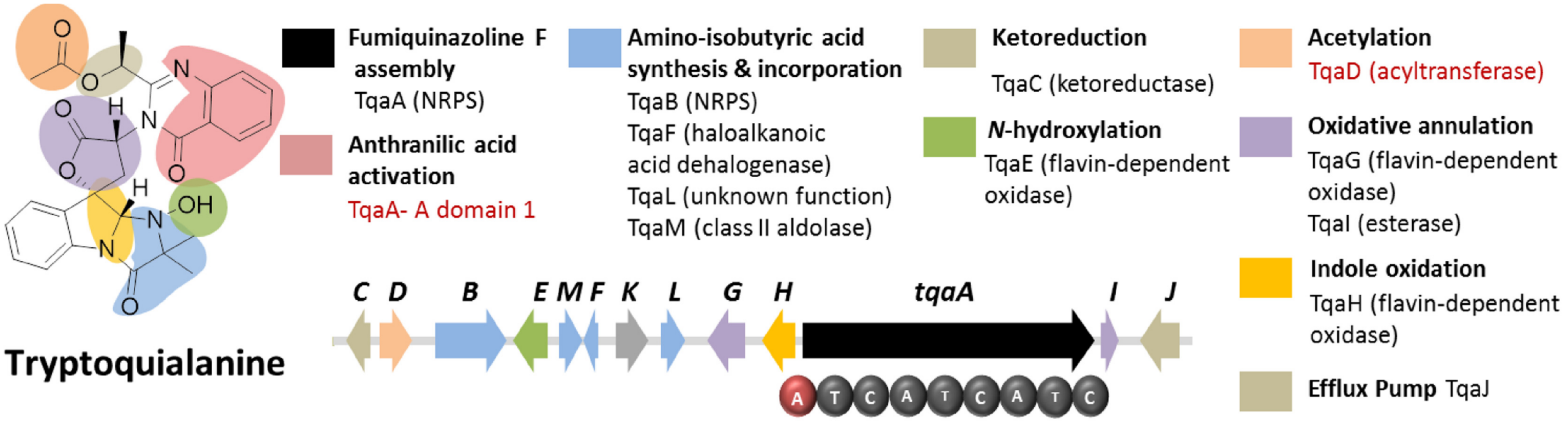
**Tryptoquialanine biosynthetic gene cluster in *P. aethiopicum.*** The presence of acyltransferase gene *tqaD* and the anthranilic acid-activating A domain of TqaA (in red text) were clues to identification of the cluster.

Tryptoquialanine (**Figure 6**) is a quinazoline-containing indole alkaloid produced by *P. aethiopicum*. It is structurally similar to a known tremorgenic mycotoxin from *A. clavatus* tryptoquivaline. Due to the presence of the non-proteinogenic aa anthranilic acid in the scaffold of the peptide, it was inferred that the compound is assembled by a NRPS (Ames and Walsh, 2010). Comparative bioinformatic analysis of the NRPS genes between *P. aethiopicum* and *P. chrysogenum* revealed the presence of 10 NRPS genes in *P. aethiopicum* that are non-orthologous to the NRPS genes in *P. chrysogenum* and were therefore candidates for tryptoquialanine biosynthesis (Gao et al., 2011). On the other hand, comparative bioinformatic analysis of the NRPS genes in *P. aethiopicum* with NRPS genes with the tryptoquivaline-producing *A. clavatus*, led to the identification of two NRPS genes in *P. aethiopicum* that are orthologous with *A. clavatus* NRPS genes but not with *P. chrysogenum* NRPS genes (**Figure 3B**). The two candidate NRPS genes, found on two separated scaffolds, encode a trimodule NRPS annotated as *tqaA* and a single-module NRPS annotated as *tqaB*. Primer walking and fosmid sequencing later revealed that both NRPS genes are co-localized in one segment of the genome. This demonstrates that cosmid library can be used to complement NGS-based targeted SM gene cluster discovery. Furthermore, the discovery of *tqa* cluster facilitated the identification of the tryptoquivaline biosynthetic genes, which are distributed on three separated genomic loci in *A. clavatus* (Gao et al., 2011).

Sequence analysis of TqaA revealed high overall shared identity with Af12080, which was previously implicated to be involved in the biosynthesis of fumiquinazoline A, a related quinazoline-containing alkaloid from *A. fumigatus* (Ames and Walsh, 2010*)*. Subsequent in-depth characterization of TqaA revealed its role in assembling fumiquinazoline F from anthranilate, L-tryptophan and L-alanine (Gao et al., 2012). The study also demonstrated the function of the terminal condensation domain in cyclization of the NRPS product and facilitated future genome mining of fungal cyclic non-ribosomal peptides. On the other hand, the second NRPS TqaB, based on the preliminary retrobiosynthetic analysis, presumably installs the 2-amino-isobutyric acid portion of the imidazolindolone pendant group (Gao et al., 2011). Interestingly, this reflects the arrangement of the aa building blocks, where anthranilic acid, L-tryptophan and L-alanine make up the main peptide chain while the fourth building block 2-amino-isobutyric acid is added to the modified L-tryptophan side chain.

**Figure 6** shows the other genes involved in the biosynthesis of tryptoquialanine (Gao et al., 2011). Based solely on the conserved domains of the enzymes encoded by the *tqaA* genes, only the function of TqaD in the installation of the acetyl group in the tryptoquialanine can be confidently assigned. The function of the remaining genes in the cluster could only be assigned by targeted deletion of individual genes (Gao et al., 2011). This revealed that *tqaE* and *tqaH* are responsible for *N*-hydroxylation and the 2,3-epoxidation of the pendant indole ring of tryptoquialanine, respectively. *tqaC* encodes a short-chain dehydrogenase that reduces the ketone in tryptoquialanone. Meanwhile, *tqaG* and *tqaI* were demonstrated to encode enzymes that mediate the formation of *N*-deoxytryptoquialanone. Finally, knockout of *tqaL* and *tqaM* demonstrated that both genes are required for the biosynthesis of the 2-amino-isobutyric acid.

### CASE STUDY D: ECHINOCANDIN B

**FIGURE 7:**
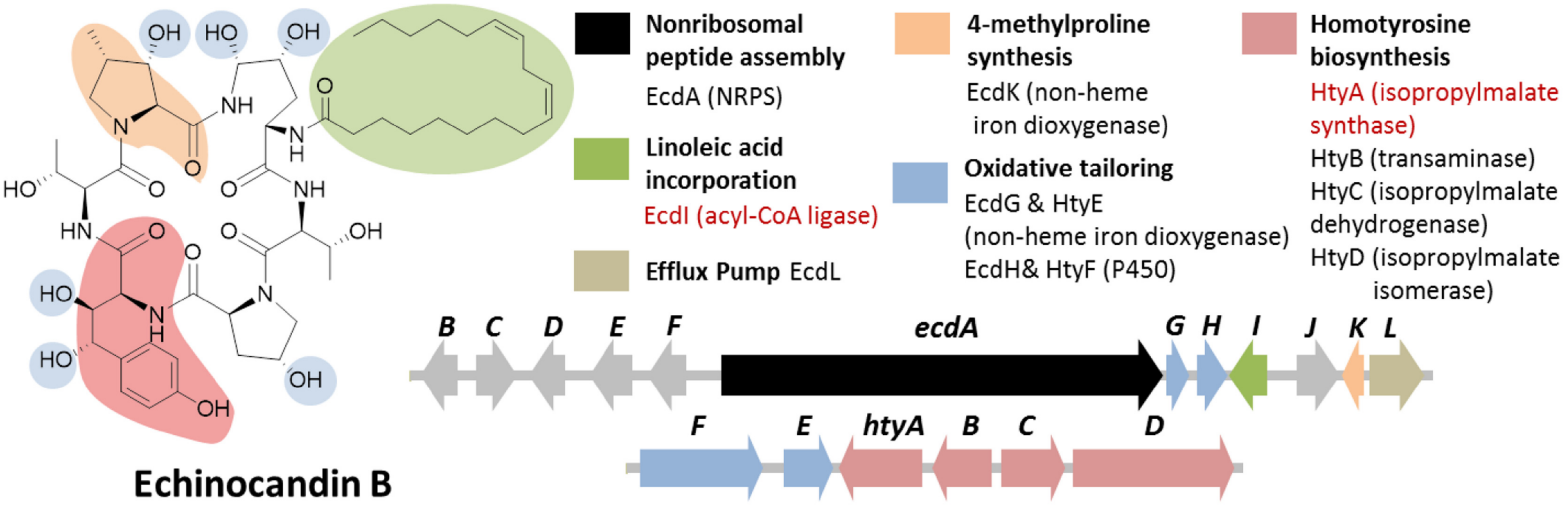
**Echinocandin B biosynthetic gene cluster in *E. rugulosa*.** The gene cluster was found in two loci (*ecd* and *hty*). The *ecd* cluster contained a six module NRPS gene *ecdA* and an acyl-CoA ligase gene *ecdI* (in red text), indicating that the gene cluster is involved in the biosynthesis of a lipo-hexapeptide SM. The separate homotyrosine biosynthesis *hty* gene cluster at a different locus was located by searching for an isopropylmalate synthase (IPMS) homolog (in red text).

Echinocandin B is a cyclic lipopeptide made by the ascomycetes *E. rugulosa.* Due to their efficacy against a broad range of *Candida* species, semisynthetic derivatives of natural echinocandins such as anidulafungin, micafungin, and caspofungin are currently in use as frontline treatment against invasive candidiasis (Kett et al., 2011). Structurally, echinocandin B is consist of six aa: (4*R*, 5*R*)-4,5-dihydroxy-L-ornithine, two units of L-threonine, (3*R*)-3-hydroxy-L-proline, (3*S*, 4*S*)-3,4-dihydroxy-L-homotyrosine, and (3*S*, 4*S*)-3-methyl-4-hydroxy-L-proline. In addition, a linoleic acid is appended to the cyclic peptide comprising of the six-aa (**Figure 7**). As with the examples given above, genome mining for the echinocandin biosynthetic gene cluster is initiated with comparative genomic analysis; in this case, between the NRPS genes in *E. rugulosa* and *A. nidulans* A4 (von Dohren, 2009; Cacho et al., 2012). Since *A. nidulans* A4 was not known to produce echinocandin B, NRPS genes orthologous to both species can be eliminated as candidates for echinocandin synthetase. Annotation of the unique NRPS genes in *E. rugulosa* revealed that only one of the ten non-orthologous NRPS genes encode for a six-module NRPS (one NRPS module is minimally consist of a C, A and T domain), which was the required number for the assembly of echinocandin B based on the collinearity rule (**Figure 3C**). In addition, the six-module NRPS, annotated as EcdA, contained an additional C-terminal condensation domain that is expected to catalyze the cyclization of the full-length peptide product (Gao et al., 2012), in accordance with the cyclic nature of the compound.

In addition to the NRPS gene *ecdA*, other genes found in the cluster in agreement to the chemical features of the compound were also found in the cluster such as the acyl-CoA ligase gene *ecdI* and the redox enzymes *ecdG, H* and *K* (**Figure 7**; Cacho et al., 2012). The presence of *ecdI*, in conjunction with its co-localization with the NRPS gene *ecdA*, was highly indicative that the product of the *ecd* gene cluster is a lipopeptide since EcdI, based on its predicted conserved domain, belongs to a family of enzyme that can convert a carboxylic acid to the more reactive acyl-CoA. Enzymatic characterization of EcdI verified its role in the activation and transfer of linoleic acid to the N-terminal thiolation domain of EcdA.

While the presence of the multiple genes for redox enzymes in the locus suggested that the product of the *ecd* gene cluster undergoes a plethora of oxidative modification steps, the low shared identity of the protein sequences of EcdG, H and K with of then characterized protein in sequence databases hindered the deciphering of the exact roles of the enzymes solely by bioinformatic analysis. Later gene knockout and enzymatic reconstitution study revealed the regioselectivity of EcdG toward the C3 of L-homotyrosine and regioselectivity of EcdH toward C4 and C5 of L-ornithine in echinocandin B biosynthesis (Jiang et al., 2013). On the other hand, EcdK was revealed to perform the two-step oxidation of L-leucine to afford 5-hydroxy-L-leucine and γ-methyl-glutamic acid-γ-semialdehyde en route to the biosynthesis of (4*R*)-*R*-methyl-L-proline (Jiang et al., 2013).

Notably missing in the *ecd* gene cluster, however, were the genes required for the biosynthesis of L-homotyrosine, another non-proteinogenic aa building block of echinocandin B. Based on previous labeling studies on L-homotyrosine biosynthesis demonstrated that the latter originated from acetate and L-tyrosine (Adefarati et al., 1991). It was also proposed that the pathway is analogous to that of L-leucine biosynthesis, the first step of which is catalyzed by isopropylmalate synthase (IPMS). Thus, we predicted that a homolog of IPMS was involved in the first step of the biosynthesis of L-homotyrosine and found by genome mining the IPMS-like gene *htyA* in the *E. rugulosa* genome that is non-orthologous to the IPMS gene in *A. nidulans* A4 genome. Subsequent deletion of *htyA* implicated its role in the biosynthesis of L-homotyrosine (Cacho et al., 2012). Flanking the *htyA* gene were additional genes presumably involved in the biosynthesis of L-homotyrosine (*htyB*, *C*, and *D*) as well as two additional oxidase genes *htyE* and *htyF*.

Shortly after the studies on echinocandin B biosynthesis were reported, the biosynthetic gene cluster for the structurally-related pneumocandin B was described (Chen et al., 2013). The pneumocandin biosynthetic gene clusters contained gene homologs echinocandin gene cluster include *ecdA*, *ecdI*, cytochrome P450 genes (e*cdH* and *htyF*), *ecdG*, *htyE*, and *ecdK* and the L-homotyrosine biosynthetic genes. Interestingly, whereas the echinocandin B biosynthetic genes are located at multiple loci, the pneumocandin biosynthetic genes are situated in a single gene cluster. In addition, the pneumocandin biosynthetic gene cluster also contained a HR-PKS gene for the biosynthesis of the 10,12-dimethyl-myristic acid and an additional 2-oxoglutarate, non-heme iron-dependent oxygenase gene (GLAREA10042) which presumably is involved in the hydroxylation of L-glutamine at C3; both corresponding to the structural features of pneumocandin B but not of echinocandin B. The recent availability of several fungal genomes that encode production of the echinocandin family of lipopeptides has allowed bioinformatics comparison of these gene clusters and enables functional prediction of the unique pathway genes in correspondence to the unique structural features of individual echinocandin analogs (Bills et al., 2014).

## CONCLUSION AND OUTLOOK

Next-generation sequencing technologies have significantly accelerated the process of targeted SM gene cluster discovery. The whole process from extraction of genomic DNA of the producing-fungus, sequencing, to initial identification of the target SM gene cluster can occur in one to three months. Using the described strategies, we can often identify the target SM gene cluster in the genome, within 2 weeks from receiving the sequencing data, with high accuracy. Compared to the traditional genomic library screening approach, which can take years beginning from the process of identifying the first biosynthetic gene to finally obtaining the complete gene cluster, such pace of discovery is unimaginable as recent as half a decade ago. The more arduous task ahead is the subsequent gene cluster verification and characterization as fungal genetic transformation protocols need to be developed for individual organisms. The development of heterologous expression systems and *in vitro* recombinant enzyme characterization methods greatly complement the genetic approach and can provide biosynthetic insights unattainable by traditional knockout approach. More and more fungal natural product research labs are expected to take advantage of NGS technology for identifying the gene cluster encoding the biosynthesis of SMs of interest. The increasing throughput and lowering cost for NGS would also encourage the simultaneous sequencing of multiple strains that produce a same family of compounds, which enables the use of powerful comparative genomics tools in the gene cluster identification and promotes combinatorial biosynthesis of fungal natural products.

## Conflict of Interest Statement

The authors declare that the research was conducted in the absence of any commercial or financial relationships that could be construed as a potential conflict of interest.

## Footnotes

1 http://www.ncbi.nlm.nih.gov/guide/howto/run-blast-local/

2 http://www.clcbio.com/

3 http://www.geneious.com/

4 http://www.softberry.com/berry.phtml?topic=index&group=programs&subgroup=gfind

5 http://bioinf.uni-greifswald.de/webaugustus/predictiontutorial.gsp#exampledata
